# An update of the *Culicoides* (Diptera: Ceratopogonidae) checklist for the Balkans

**DOI:** 10.1186/s13071-018-3051-x

**Published:** 2018-08-13

**Authors:** Dubravka Pudar, Dušan Petrić, Xavier Allène, Bulent Alten, Nazlı Ayhan, Aleksandar Cvetkovikj, Claire Garros, Teufik Goletić, Filiz Gunay, Kristyna Hlavackova, Aleksandra Ignjatović Ćupina, Mihaela Kavran, Tereza Lestinova, Bruno Mathieu, Ognyan Mikov, Igor Pajović, Ignace Rakotoarivony, Jovana Stefanovska, Slavica Vaselek, Almedina Zuko, Thomas Balenghien

**Affiliations:** 1 Faculty of Agriculture, Department of Phytomedicine and Plant Protection, Laboratory for Medical and Veterinary Entomology, University of Novi Sad, Novi Sad, Serbia; 2 CIRAD, UMR ASTRE, F-34398 Montpellier, France; 3 ASTRE, University Montpellier, CIRAD, INRA, Montpellier, France; 4 Faculty of Science, Department of Biology, Ecology Division, VERG Laboratories, Hacettepe University, Beytepe-Ankara, Turkey; 5 Virology Unit, Faculty of Medicine, Aix-Marseille University, Marseille cedex 05, France; 6 Faculty of Veterinary Medicine, Department of Parasitology and Parasitic Diseases, Ss. Cyril and Methodius University in Skopje, Skopje, Republic of Macedonia; 7 CIRAD, UMR ASTRE, F-97490 Sainte Clotilde, Réunion; 8 Veterinary Faculty, Department of Zootechnics and Poultry, University of Sarajevo, Sarajevo, Bosnia and Herzegovina; 9 Faculty of Science, Department of Parasitology, Charles University in Prague, 2 Prague, Czech Republic; 10 Medicine Faculty, Institute of Parasitology and Tropical Pathology, University of Strasbourg, EA7292 Strasbourg, France; 11 National Centre of Infectious and Parasitic Diseases, Department of Parasitology and Tropical Medicine, Laboratory of Experimental and Applied Parasitology, Sofia, Bulgaria; 12 Biotechnical Faculty, University of Montenegro, Podgorica, Montenegro; 13 Veterinary Faculty, Department of Parasitology and Invasive Diseases, University of Sarajevo, Sarajevo, Bosnia and Herzegovina; 14 IAV Hassan II, MIMC unit, Rabat, Morocco

**Keywords:** *Culicoides*, New records, Checklist, Balkans

## Abstract

**Background:**

The prime significance of species belonging to the genus *Culicoides* Latreille, 1809 (Diptera: Ceratopogonidae) is their ability to transmit viruses such as bluetongue virus (BTV) to wild and domestic ruminants. Prior to 1998, BTV was considered exotic in Europe, but according to recent history of its outbreaks, it has become endemic in southern and eastern European countries circulating beyond its expected historical limits, into the Balkan region. The wind-borne long-distance dispersal of *Culicoides* spp. over water bodies and local spreading between farms emphasize the necessity of filling in the information gaps regarding vector species distribution. In most Balkan countries, data on *Culicoides* fauna and species distribution are lacking, or information is old and scarce.

**Results:**

During this study, 8586 specimens belonging to 41 species were collected. We present the first faunistic data on *Culicoides* species in the former Yugoslav Republic of Macedonia (FYROM), Kosovo, Montenegro and Serbia. For other countries (Bosnia and Herzegovina, Bulgaria and Croatia), all historical records were compiled for the first time and then expanded with our findings to various extents. In all countries, confirmed or suspected BTV vector species belonging to the subgenera *Avaritia* and *Culicoides* were collected. The total number of species sampled during our field collections was 20 in Bosnia and Herzegovina (15 new records), 10 in Bulgaria (2 new records), 10 in Croatia (5 new records), 13 in FYROM, 9 in Kosovo, 15 in Montenegro, and 28 in Serbia. Of these, 14 species were registered for the first time in this part of the Balkans.

**Conclusions:**

This paper provides the first data about *Culicoide*s fauna in FYROM, Kosovo, Montenegro and Serbia, as well as new records and an update on the checklists for Bosnia and Herzegovina, Bulgaria and Croatia. These findings provide preliminary insights into the routes of BTV introduction and spreading within the Balkans, and present a valuable contribution to further research related to *Culicoides*-borne diseases in Europe.

## Background

Biting midges of the genus *Culicoides* Latreille, 1809 (Diptera: Ceratopogonidae) are among the smallest hematophagous dipteran insects, usually 1–3 mm in body length, including at least 1368 extant species [1, 2].

Although *Culicoides* spp. cause a significant biting nuisance (impacting tourism and forestry) [3–5], their greatest importance lies in the fact that they are biological vectors of human and animal pathogens (mainly viruses, but also protozoans and filarial nematodes) [1, 5]. Indeed, the prime significance of *Culicoides* spp. is their ability to transmit viruses of domestic ruminants, such as bluetongue virus (BTV) or the novel Schmallenberg virus, which was first detected in 2011 in Germany [6].

Bluetongue (BT), which has to be reported to the Office International des Épizooties (OIE) [1, 7], is caused by BTV, affecting domestic and wild ruminants, in particular certain breeds of sheep (causing severe clinical disease and in some cases significant mortality) [8], but also cattle [9] and goats [10]. Bluetongue causes considerable economic concern and poses a major risk in the international trade of animals and animal products [11].

Prior to 1998, the disease was considered exotic in Europe [11], with just a few sporadic incursions recorded in southern European countries (Spain, Portugal, Greece and Cyprus) [12–14], but the situation has since dramatically changed. In the seven-year period from 1998 to 2005, BTV outbreaks were recorded in 15 southern European countries [11] and followed by sudden and unexpected outbreaks in northern Europe during 2006–2008 [14–17]. According to the European Food Safety Authority (EFSA) [8], this was arguably the largest and the most continuous outbreak of a *Culicoides*-borne pathogen in the history of humankind and caused enormous economic losses. Interestingly, BTV serotypes affecting southern and northern Europe are different [14]. Thanks to the implementation of compulsory BT vaccination in Europe resulting in a massive reduction of BTV cases, outbreaks were limited and some countries were back to a free BTV status.

When observing the situation in the Balkans, BTV-9 first entered southeastern Europe (Greece) in 1998 [18]. During 1999, this serotype spread from Greece to southeastern Bulgaria [19], while further outbreaks caused by BTV-9 were reported during 2001 in the former Yugoslav Republic of Macedonia (FYROM) [20], Kosovo [21], Montenegro, Serbia [22, 23] and Croatia [24, 25]. During the following year (2002), the BTV-9 outbreak was confirmed in Bosnia and Herzegovina [26] and Albania [27]. Interestingly, in the following years other serotypes of the virus were isolated in the Balkans: BTV-16 in Croatia during 2004, and an important BTV-4 epizootic in Greece, Bulgaria, Croatia, Serbia, Montenegro and FYROM in the period 2014–2017 [18].

According to the recent BT outbreak records in Europe, it is reasonable to state that BT is endemic in the countries of southern and eastern Europe and that different serotypes of BTV are still circulating through Europe.

Bluetongue emergence in previously unaffected regions of the Mediterranean basin has been attributed to climate change, through an increasing abundance of *Culicoides imicola* Kieffer, 1913, a proven Afrotropical vector species [28, 29]. However, this was not applicable to the Balkan region as *C. imicola* did not occur (except in Greece) where BT outbreaks were recorded [11, 30–32]. Meanwhile, the Palaearctic species belonging to the subgenera *Avaritia* and *Culicoides* were found implicated as BTV vector species in different European countries [11, 33].

Laboratory and field studies reported the following species as probable BTV vectors: (i) subgenus *Avaritia*: *Culicoides obsoletus* (Meigen, 1818); *Culicoides scoticus* Downes & Kettle, 1952; *Culicoides dewulfi* Goetghebuer, 1936; and *Culicoides chiopterus* (Meigen, 1830); (ii) subgenus *Culicoides*: *Culicoides pulicaris* (Linnaeus, 1758); *Culicoides punctatus* (Meigen, 1804); and *Culicoides newsteadi* Austen, 1921 [33–37]. In addition, Goffredo et al. [37] reported positive pools of the “Nubeculosus complex” [including *Culicoides nubeculosus* (Meigen, 1830), *Culicoides puncticollis* (Becker, 1903) and *Culicoides riethi* Kieffer, 1914] during the 2012–2014 BT epidemics in Italy, and recommended further studies to understand the possible vector role of these species.

Although the distribution or presence of *Culicoides* spp. are well studied in western Europe [38–41], published data on *Culicoides* fauna and species distribution for most Balkan countries are limited and there are no recent studies. To our knowledge, there are no published data for FYROM and Montenegro. In the late 1970s, Callot & Kremer [42] carried out a few collections in former Yugoslavia, recording ten *Culicoides* spp. in Bosnia and Herzegovina and five *Culicoides* spp. in Croatia. Recently, new records were published in Bosnia and Herzegovina and in Kosovo. Reporting *Culicoides obsoletus* using morphological identification (wing pattern only) raises the question about the presence of this species or its sympatric related species (*C. scoticus*) [43], and describing identification at the species “complex” level [44], both bring up taxonomic concerns.

In Serbia, Pavlović et al. [45] reported four species (*C. obsoletus*, *C. pulicaris*, *Culicoides parroti* Kieffer, 1922 and *C. nubeculosus*) in a conference abstract, but the lack of details on the identification method does not allow an assessment as to whether these authors were referring to species or groups of species. Comprehensive checklists are available only for Croatia with 16 species [42, 46, 47], for Albania with 20 species [48] and for Bulgaria with 37 species [49, 50], for which Bobeva et al. [49] summed up previously published literature, mostly in Bulgarian language [51–53].

We assembled all *Culicoides* species records from the region, combining the data collected during field studies performed by several research groups and the historical records (for Bosnia and Herzegovina, Bulgaria and Croatia). In addition, we provide the first checklists for *Culicoides* species for FYROM, Kosovo, Montenegro and Serbia.

## Methods

### *Culicoides* sampling

**Fig. 1:**
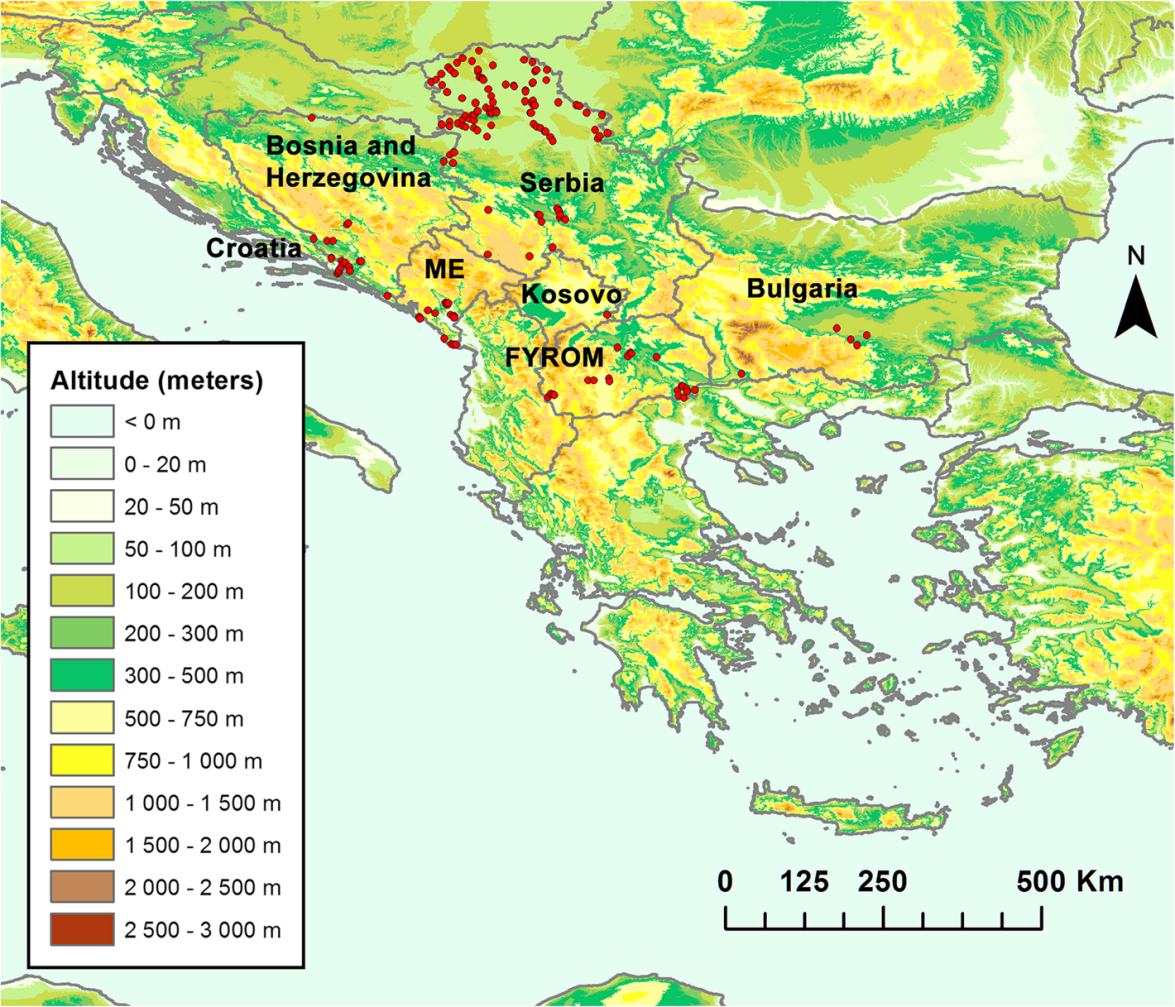
*Culicoides* collection sites in the study area. A background elevation map was created with data produced by Jonathan de Ferranti and downloaded from viewfinderpanoramas.org. *Abbreviations*: ME, Montenegro; FYROM, the former Yugoslav Republic of Macedonia

*Culicoides* spp. collections in the study area (Fig. 1) were conducted during sand fly (Diptera: Psychodidae: Phlebotominae) and/or mosquito (Diptera: Culicidae) surveys. Therefore, the selection of sites and trap types was not specifically adjusted to *Culicoides* trapping, except once in Serbia when an Onderstepoort Veterinary Institute blacklight suction trap (OVI trap) was used [54]. All traps were run for one night and set up from approximately 1 h before sunset to 1 h after sunrise.

In Bosnia and Herzegovina, 18 sites were surveyed over two periods. Five sites were sampled during a mosquito survey (17–22/06/2015) by NS2 dry ice-baited traps without light (constructed at the University of Novi Sad, Serbia), while another 13 sites were prospected during sand fly sampling (03–07/07/2015) using standard miniature Centre for Disease Control (CDC) light traps (John W. Hock, model 512, Gainesville, FL, USA) without dry ice.

In Bulgaria, 5 sites were sampled during sand fly collections (20–24/08/2015) using miniature CDC light traps with and without dry ice.

In Croatia, miniature CDC light traps without dry ice were used, during sand fly field studies (13–15/07/2015) at two sites.

In FYROM, 21 sites were sampled during a sand fly campaign (24/08/2015–14/09/2015) using miniature CDC light traps with and without dry ice.

In Kosovo, one site was surveyed (01–02/09/2015) using miniature CDC light traps without dry ice.

In Montenegro, specimens were obtained from 25 sites (urban, semi-urban and rural areas) during mosquito sampling (16–21/05/2015) by NS2 and BG-Sentinel traps (Biogents, Regensburg, Germany) baited with dry ice.

In Serbia, collections were undertaken at 124 sites (urban, semi-urban and rural areas) (28/04/2015–13/09/2015) during a West Nile virus surveillance program (81 sites, using NS2 traps only) and sand fly collections (43 sites). Within these 43 sites, 14 sites were exclusively sampled for sand fly collections and only CDC light traps without dry ice were used; at the other 29 sites, NS2 and miniature CDC light traps with and without dry ice were used and an OVI trap was placed only once. At these 29 sites, all mentioned trap types were sometimes positioned to potentiate the collection of *Culicoides* as well (depending on the number of animals and their composition, as well as farmers’ permission for trap placing). According to the study context, the traps were placed bi-weekly, monthly or just once per collecting season in different parts of Serbia.

*Culicoides* biting midges in other surveyed countries were collected in traps positioned to sample sand flies (all traps in Bulgaria, Croatia, FYROM, Kosovo, and 72% of the traps placed in Bosnia and Herzegovina) and/or mosquitoes (all traps in Montenegro and 28% of the traps in Bosnia and Herzegovina). In all countries, the sand fly surveys were carried out in farms (usually containing different mammal species and much rarely avian species). During the mosquito surveys, traps were positioned in different kinds of sites: highly urban (without any animal breeding); semi-urban (with different animal breeding); and rural (far away from human settlements and farms).

All sand fly-specific sites gained *Culicoides* spp. positive samples. Consequently, in the countries where only sand fly monitoring was performed, frequency of occurrence of different *Culicoides* spp. is expressed as a ratio of positive to all collection sites. As the sites for mosquito sampling were chosen according to different criteria, usually not comprising the courtyards, *Culicoides* spp. were not collected at all of them. Therefore, for Montenegro and Serbia, only the *Culicoides* spp. positive mosquito sampling sites were considered for the frequency of occurrence evaluation.

### *Culicoides* identification

Species identification was carried out by observing morphological features under a stereomicroscope. Primarily, we focused on the wing pigmentation pattern and the distribution of wing macrotrichia. Then, we observed the antennal XI/X ratio (length of segment XI divided by length of segment X), and the shape and size of the 3rd palpal segment. Finally, we compared all observed traits with IIKC (interactive identification key for *Culicoides*) database pictures [55]. Slide mounting was performed in the cases of morphologically similar species, atypical variations of the wing pattern, identification confirmation, or if the specimen was damaged. Slide-mounted specimens were observed under a light microscope connected to a camera and attention was paid to the broad range of other morphological features including shape, size and the number of female spermathecae, the shape and form of male genitalia [56, 57], as well as eye separation distance [55].

*Culicoides obsoletus* females cannot be morphologically separated with confidence from *C. scoticus* females or can be distinguished only by using many morphological measurements [58–60]. Thus, in our paper, we refer to *C. obsoletus*/*C. scoticus* females*.* However, males were recorded as *C. obsoletus* or *C. scoticus*, because they can be distinguished based on the appearance of the genitalia [55]. The *Culicoides obsoletus* taxon refers to *C. obsoletus* (*sensu lato*), i.e. potentially including the cryptic species, which have been recently discovered [61, 62].

In some cases, morphologically closely related species, or specimens with an atypical wing pattern (i.e. variations and overlapping between two species) could not be morphologically separated and identified with confidence. We refer to these as pairs of species, such as *Culicoides fagineus* Edwards, 1939*/Culicoides subfagineus* Delécolle & Ortega, 1998; *Culicoides lupicaris* Downes & Kettle, 1952*/Culicoides pulicaris*; *Culicoides cataneii* Clastrier, 1957*/Culicoides gejgelensis* Dzhafarov, 1964; and *Culicoides jumineri* Callot & Kremer, 1969*/Culicoides kurensis* Dzhafarov, 1960. *Culicoides lupicaris* was morphologically identified as described by Delécolle [63] since a formal redescription is missing [64]. We decided to associate *sensu lato* to *Culicoides grisescens* Edwards, 1939 to take into account the cryptic diversity highlighted recently within this taxon [61]. It is also worth noting that females of this species could be confused with *Culicoides deltus* Edwards, 1939. Although according to Borkent’s catalog, *Culicoides trivittatus* Vimmer, 1932 belongs to the subgenus *Oecacta* [65], we decided to place it in “Miscellaneous unplaced species” (Table 1) since the male genitalia are very particular and very different from the type-species *Oecacta furens* [66]. However, in numerous trapping sites, the morphological identification to the species level of females and of males (confirmed by the observation of their species-specific genitalia appearance) was possible, providing us with information on the records of the members of the species complexes in most countries.

**Table 1:** Checklist of *Culicoides* records in Croatia, Bosnia and Herzegovina, Serbia, Montenegro, Kosovo, FYROM and Bulgaria

*Culicoides* species	Croatia		Bosnia and Herzegovina		Serbia		Montenegro		Kosovo		FYROM		Bulgaria	
	Pres	Ref	Pres	Ref	Pres	Ref	Pres	Ref	Pres	Ref	Pres	Ref	Pres	Ref
*Avaritia* Fox					×		×		×		×			
*Culicoides chiopterus* (Meigen, 1830)									×					
*Culicoides dewulfi* Goetghebuer, 1936													×	[49]
*Culicoides obsoletus*/*Culicoides scoticus*	×		×	[43]^a^	×		×		×		×		×	
*Culicoides obsoletus* (Meigen, 1818)	*	[46, 47]			×		×						*	[49]
*Culicoides scoticus* Downes & Kettle, 1952	*	[46, 47]					×						*	[49]
*Beltranmyia* Vargas					×		×							
*Culicoides circumscriptus* Kieffer, 1918	*	[42, 46, 47]	×	[42]	×		×						*	[49]
*Culicoides manchuriensis* Tokunaga, 1941					×									
*Culicoides salinarius* Kieffer, 1914			×		×								*	[49]
*Culicoides* Latreille			×		×		×		×		×			
*Culicoides deltus* Edwards, 1939													*	[49]
*Culicoides fagineus*/*Culicoides subfagineus*	×		×						×				×	
*Culicoides fagineus* Edwards, 1939	*	[46]			×								*	[49]
*Culicoides subfagineus* Delécolle & Ortega, 1998					×									
*Culicoides flavipulicaris* Dzhafarov, 1964					×						×		×	[49]
*Culicoides grisescens* Edwards, 1939 (*s.l.*)			×								×		×	[49]
*Culicoides impunctatus* Goetghebuer, 1920													*	[49]
*Culicoides lupicaris*/*Culicoides pulicaris*					×									
*Culicoides lupicaris* Downes & Kettle, 1952			×						×				×	
*Culicoides pulicaris* (Linnaeus, 1758)	*	[46]	×		×				×		×		×	[49]
*Culicoides newsteadi* Austen, 1921	×	[47]	×		×		×		×		×		×	[49]
*Culicoides punctatus* (Meigen, 1804)	×	[46]	×		×		×		×		×		×	[49]
*Monoculicoides* Khalaf					×				×		×			
*Culicoides nubeculosus* (Meigen, 1830)					×				×				*	[49]
*Culicoides parroti* Kieffer, 1922													*	[49]
*Culicoides puncticollis* (Becker, 1903)					×								×	[49]
*Culicoides riethi* Kieffer, 1914					×						×		*	[49]
*Culicoides stigma* (Meigen, 1818)													*	[49]
*Oecacta* Poey					×		×				×			
*Culicoides brunnicans* Edwards, 1939					×									
*Culicoides dzhafarovi* Remm, 1967			*	[42]										
*Culicoides longipennis* Khalaf, 1957	*	[42]	×	[42]	×						×		*	[49]
*Culicoides picturatus* Kremer & Deduit, 1961			×		×		×							
*Culicoides saevanicus* Dzhafarov, 1960	*	[46]												
*Culicoides vexans* (Staeger, 1839)													*	[49]
*Pontoculicoides* Remm														
*Culicoides saevus* Kieffer, 1922													*	[49]
*Culicoides sejfadinei* Dzhafarov, 1958			*	[42]									*	[49]
*Culicoides tauricus* Gutsevich, 1959													*	[49]
*Remmia* Glukhova														
*Culicoides schultzei* (Enderlein, 1908)													*	[49]
*Sensiculicoides* Shevchenko					×		×				×			
*Culicoides alazanicus* Dzhafarov, 1961			*	[42]^b^	×		×						*	[50]
*Culicoides begueti* Clastrier, 1957			×				×							
*Culicoides cataneii* Clastrier, 1957/*Culicoides gejgelensis* Dzhafarov, 1964	×	[42]	×	[42]	×		×				×		*	[49]
*Culicoides clastrieri* Callot, Kremer & Deduit, 1962			*	[42]										
*Culicoides duddingstoni* Kettle & Lawson, 1955					×									
*Culicoides festivipennis* Kieffer, 1914	×		×	[42]^c^	×		×				×		*	[49]
*Culicoides griseidorsum* Kieffer, 1918	×		×		×		×							
*Culicoides haranti* Rioux, Descous & Pech, 1959	*	[46]												
*Culicoides indistinctus* Khalaf, 1961													×	
*Culicoides jumineri* Callot & Kremer, 1969/*Culicoides kurensis* Dzhafarov, 1960	×		×										*	[49]
*Culicoides kibunensis* Tokunaga, 1937	*	[42]^d^	*	[42]^d^	×		×				×			
*Culicoides maritimus* Kieffer, 1924	*	[47]												
*Culicoides odiatus* Austen, 1921					×								*	[49]
*Culicoides pictipennis* (Staeger, 1839)							×						*	[49]
*Culicoides shaklawensis* Khalaf, 1957											×		*	[49]
*Culicoides simulator* Edwards, 1939					×								*	[49]
*Culicoides submaritimus* Dzhafarov, 1962			×											
*Culicoides vidourlensis* Callot, Kremer, Molet & Bach, 1968	*	[42]	*	[42]										
*Silvaticulicoides* Glukhova			×		×		×		×		×			
*Culicoides achrayi* Kettle & Lawson, 1955					×		×							
*Culicoides fascipennis* (Staeger, 1839)	×	[46]	×						×		×		*	[49]
*Culicoides ostroushkoae* Glukhova, 1989													*	[49]
*Culicoides pallidicornis* Kieffer, 1919					×								*	[49]
*Culicoides subfasciipennis* Kieffer, 1919			×		×								*	[49]
*Wirthomyia* Vargas			×		×		×							
*Culicoides minutissimus* (Zetterstedt, 1855)			×		×		×							
*Culicoides reconditus* Campbell & Pelham-Clinton, 1960													*	[49]
Miscellaneous unplaced species														
*Culicoides trivittatus* Vimmer, 1932	×													
*Culicoides paolae* Boorman, 1996	*	[46]												

*Abbreviations*: *Pres*, presence; *Ref*, reference; *FYROM*, the former Yugoslav Republic of Macedonia

^a^Reported as *Culicoides obsoletus* but identified only by wing morphology

^b^Reported as *Culicoides musilator* Kremer & Callot, 1961

^c^Reported as *Culicoides odibilis* Austen, 1921

^d^Reported as *Culicoides cubitalis* Edwards, 1939

*Key*: ×, the first record of certain species or subgenera in the country; ×, species recorded in previous studies and the present study; *, species recorded by previous authors

The subgenera and species are classified and presented in accordance with Borkent [65], Mathieu et al. [55], Ramilo et al. [64] and Szadziewski et al. [66]. *C. indistinctus* is presented in Borkent [65] as a synonym of *C. odiatus. C. submaritimus* is presented in Borkent [65] as a synonym of *C. maritimus.* Bobeva et al. [49] summed up previously published literature for Bulgaria, citing: Zilahi [51], Remm [52] and Nedelchev [53]

Furthermore, only a few specimens, too damaged to be assigned to a given species, were classified as *Culicoides* sp.

## Results

A total of 8586 *Culicoides* specimens belonging to at least 41 species (at least 40 species classified in eight subgenera plus 1 miscellaneous unplaced species [55, 64–66]) were processed during our study (Table 1). Species of an additional two subgenera were recorded by previous authors (Table 1).

Only *C. punctatus*, *C. newsteadi* and *C. obsoletus/C. scoticus* were recorded in all inspected countries during our study. Three patterns of species presence/absence and frequency of occurrence were observed: the first in Bosnia and Herzegovina, Bulgaria, Croatia, FYROM and Kosovo; the second in Montenegro and the third, an intermediate pattern, in Serbia.

In Bosnia and Herzegovina, Bulgaria, Croatia, FYROM and Kosovo, the most frequent species were *C. punctatus* (recorded at 66% of sites), *C. newsteadi* (57%) and *C. obsoletus/C. scoticus* (38%), followed by *C. pulicaris* (30%), *Culicoides fascipennis* (Staeger, 1839) (19%) and *C. fagineus*/*C. subfagineus* (11%)*.* The less common species were *Culicoides festivipennis* Kieffer, 1914, *C. lupicaris* and *C. cataneii*/*C. gejgelensis*, while the remaining species were very rare. It should be emphasized that *C. trivittatus* was identified in Croatia; this represents the first record of the species in Europe.

The most frequent species recorded in Montenegro were *Culicoides griseidorsum* Kieffer, 1918 (recorded at 40% of sites) and *C. festivipennis* (24%), followed by *Culicoides alazanicus* Dzhafarov, 1961, *Culicoides pictipennis* (Staeger, 1839) and *C. newsteadi* (16% each). *Culicoides punctatus* and *Culicoides minutissimus* (Zetterstedt, 1855) were rare (12% each). Records of other species (including *C. obsoletus/C. scoticus*) were negligible, whereas *Culicoides pulicaris* was not found.

The situation in Serbia seemed to be intermediate. *Culicoides festivipennis* was convincingly the most frequent species (recorded at 59% of sites), followed by *Culicoides circumscriptus* Kieffer, 1918 and *C. griseidosum* (51% and 49%, respectively)*. Culicoides obsoletus/C. scoticus* was recorded at 30% of sites, while *C. punctatus* and *C. newsteadi* were each found at 25% of the sites. *Culicoides minutissimus*, *C. alazanicus*, and *C. pulicaris* were less frequent, observed at 21%, 16% and 9% of sites, respectively. All other species were recorded at less than 8% of sites.

In the north of Serbia, more species were recorded during the sand fly surveys (27 spp.) than during the mosquito surveys (16 spp.), even if sampling was carried out in more sites and more frequently during mosquito surveys than sand fly surveys. Only one species, *Culicoides kibunensis* Tokunaga, 1937, was collected only during the mosquito surveys and not during the sand fly surveys. Finally, *C. fagineus* was recorded in the south-west of the country where only sand fly targeted sampling was performed. Additionally, in the north of Serbia where both mosquito and sand fly surveys were carried out, *Culicoides* were found in all sites sampled for sand flies, and only in 83% of the sites (67 out of 81) sampled for mosquitoes.

The total number of species recorded during this study was as follows: at least 20 in Bosnia and Herzegovina, at least 10 in both Bulgaria and Croatia, at least 13 in FYROM, at least 9 in Kosovo, at least 15 in Montenegro and at least 28 in Serbia (Table 1).

## Discussion

This paper provides the first *Culicoides* faunistic data for FYROM, Kosovo, Montenegro and Serbia, and an update of the *Culicoides* species checklists by adding several newly recorded species: at least 15 for Bosnia and Herzegovina, two for Bulgaria, and at least five for Croatia (Table 1), as a preliminary and mandatory step prior to any further research related to *Culicoides*-borne diseases in this region of Europe.

Until recently, there was very limited information about *Culicoide*s species in the territory of former Yugoslavia. In a short note format article, Callot & Kremer [42] reported 12 *Culicoides* species based on adult emergence from mud samples collected in 1972, mainly along the coast of the Adriatic Sea (including present Bosnia and Herzegovina and Croatian territories). Additional studies in Bosnia and Herzegovina [43] and Croatia [46, 47] (Table 1) were carried out several decades later after this region was affected by a BTV outbreak [24–26], which acted as a trigger for *Culicoides* survey (re)starting. The same applies to Kosovo, where the “Obsoletus” and “Pulicaris” species complexes were reported, but without identification down to the species level [44], while for Serbia, due to the limited amount of published information (conference abstract) [45], it was not clear if the taxa mentioned were referring to species or to groups of species. Hence, we decided not to include these findings in the Serbian species checklist provided here. The research on *Culicoides* in Bulgaria has a much longer tradition and had been performed by several authors [49–53] (Table 1).

There were no previously published data on *Culicoides* in Montenegro. We recorded at least 15 species, but potential BTV vectors, such as *C. obsoletus/C. scoticus* or *C. punctatus*, which predominantly feed on large mammals [67] were very rare (only in a few localities), while *C. newsteadi* was present in more collection sites. The most frequently recorded species were *C. griseidorsum*, *C. festivipennis*, *C. alazanicus*, *C. pictipennis* and *C. newsteadi*. The species fauna was different compared to other countries surveyed in this study, which might be explained by the specific positioning of the traps aimed to sample mosquitoes. This sometimes may include rural locations (far away from human settlements and farms) or highly urban areas (without any cattle breeding). The two most common species found in Montenegro, namely *C. griseidorsum* and *C. festivipennis*, are known as predominant bird feeders [67], as well as *C. alazanicus* which was the third common species in our survey. *Culicoides alazanicus* shared the third position with *C. pictipennis*, which is an opportunistic feeder, and with *C. newsteadi*, which feeds exclusively on large mammals [50, 67]. *Culicoides* specimens were found in 28% of the traps positioned in urban sites, 64% in semi-urban sites and 8% in rural sites. Although semi-urban sites (with animal breeding) were more frequently sampled, the number of mammals was usually low (≤ 2 in 69% of cases).

In the majority of countries, contrary to the study in Montenegro, the trap locations were selected mainly to meet requirements for sand fly sampling, i.e. all collection sites in Bulgaria, Croatia, FYROM and Kosovo, as well as 72% of the traps placed in Bosnia and Herzegovina. Finally, in Serbia, 11% of the traps were set up exclusively for sand fly sampling, 24% of the collection sites were chosen for both sand fly and *Culicoides* sampling, while 65% of the traps were intended to mosquito survey. The traps set up for mosquitoes and collecting *Culicoides* were positioned in urban (15%), semi-urban (66%) and rural (19%) sites.

Although *Culicoides*, sand flies and mosquitoes can be very often found in the same environment, the choice of the trap location (particularly presence of suitable hosts nearby) can influence the species composition of each group. Since all sand fly samplings were carried out on farms (usually containing different mammal species and, much more rarely, avian species), it is not surprising that *Culicoides* catches in surveyed countries other than Montenegro and Serbia were dominated by mammophilic species: *C. punctatus*, *C. newsteadi*, *C. obsoletus/C. scoticus* and *C. pulicaris*.

In contrast, in Serbia, where 65% of the traps were placed for mosquito sampling (including urban, semi-urban and rural areas), the species composition was dominated by predominately ornithophilic species, namely *C. festivipennis*, *C. circumscriptus* and *C. griseidorsum*, followed by predominately mammophilic feeders, namely *C. obsoletus/C. scoticus*, *C. newsteadi* and *C. punctatus* [67]. This kind of species composition and the occurrence frequency of *Culicoides* spp. indicates strong correlation with the host species present at each sampling site (the selection was tailor-made for the study purpose). Moreover, the sites sampled for sand flies may be more suitable for *Culicoides* inventory than those sampled for mosquitoes because we recorded more species in the first than in the second ones. Indeed, all sites sampled for sand flies were positive for *Culicoides*, whereas 17% of the sites sampled for mosquitoes were negative (when comparing catches in the north of Serbia). It should be noted, however, that these are preliminary estimations because the number of sampling sites and the sampling frequency were not the same for the different protocols.

Out of at least 41 species of *Culicoides* collected during our study, 14 species were recorded for the first time in this part of the Balkans (Table 1). Moreover, the finding of *C. trivittatus* in Croatia represents the first record of this species in Europe. This species is known to be distributed throughout Asia and middle Asia (Israel, Iran, Iraq, Armenia, Azerbaijan, Uzbekistan, Tajikistan, Turkmenistan and the south 50°N in Russia and Ukraine) [52].

## Conclusions

We present the first data on *Culicoides* fauna in FYROM with at least 13 species, in Kosovo with at least 9 species, in Montenegro with at least 15 species and in Serbia with at least 28 species. For the other countries all previous *Culicoides* records (published independently by different author groups) were compiled for the first time and then expanded with our findings: at least 15 newly recorded species for Bosnia and Herzegovina, two for Bulgaria, and at least five for Croatia. Fourteen species were detected for the first time in the seven Balkan countries. Among the newly recorded species, *C. trivittatus* was recorded for the first time in Europe. Furthermore, due to sampling design, insights about species occurrence outside farm environments were provided. This can also serve as good starting point for research on ornithophilic *Culicoides* species, as well as surveillance of the bird pathogens they can transmit. In all countries, species of the subgenera *Avaritia* and *Culicoides*, which are proven or probable BTV vectors, were registered. This finding provides preliminary insights into the routes of BTV introduction and spreading within the Balkans.

## Abbreviations

BT: bluetongue
BTV: bluetongue virus
CDC: Centre for Disease Control
FYROM: the former Yugoslav Republic of Macedonia
ME: Montenegro
OIE: Office International des Épizooties
OVI: Onderstepoort Veterinary Institute
